# Overcoming Challenges in the Determination of Fatty Acid Ethyl Esters in Post-Mortem Plasma Samples with the Use of Targeted Metabolomics and the Quality by Design Approach

**DOI:** 10.3390/biomedicines13071688

**Published:** 2025-07-10

**Authors:** Joanna Dawidowska, Julia Jacyna-Gębala, Renata Wawrzyniak, Michał Kaliszan, Michał Jan Markuszewski

**Affiliations:** 1 Department of Biopharmaceutics and Pharmacodynamics, Medical University of Gdańsk, 80-416 Gdańsk, Poland; 2 Department of Forensic Medicine, Medical University of Gdańsk, 80-204 Gdańsk, Poland

**Keywords:** targeted metabolomics, fatty acid ethyl esters, quality by design, robustness, post-mortem plasma samples

## Abstract

**Background:** Excessive alcohol consumption constitutes a serious cause of death worldwide. Fatty acid ethyl esters, as metabolites of the non-oxidative elimination pathway of ethanol, have been recognized as mediators of alcohol-induced organ damage. These metabolites serve as potential biomarkers for the assessment of ethanol intake and might be also used in post-mortem studies. **Methods:** In this study, the development and optimization of a simple, fast, precise, accurate, and cost-effective method with the use of gas chromatography coupled with tandem mass spectrometry for quantitative analysis of six fatty acid ethyl esters, namely ethyl laurate, myristate, palmitate, linoleate, oleate, and stearate, were conducted. **Results:** The optimized method was fully validated according to ICH guidelines. Additionally, identification of critical method parameters was possible by using the quality by design approach. By carrying out analyses according to the Plackett–Burman plan (design of experiments methodology), the robustness of the analytical method developed was confirmed for four (ethyl palmitate, linoleate, oleate, and stearate) ethyl esters. In the case of ethyl myristate, the variable significantly affecting the results appeared to be the temperature of solvent evaporation after the deproteinization step. **Conclusions:** Biochemical interpretation of the obtained results with available medical records suggests that plasma concentrations of selected fatty acid ethyl esters are valuable indicators of pre-mortem alcohol consumption and may be one of the key factors helpful in determining the cause and mechanism of death.

## 1. Introduction

Excessive alcohol consumption caused 3 million deaths (5.3% of all deaths) in 2016 worldwide. Importantly, its impact on mortality is greater than that of tuberculosis (2.3%), HIV/AIDS (1.8%), diabetes (2.8%), hypertension (1.6%), gastrointestinal disease (4.5%), road traffic injuries (2.5%), and violence (0.8%), all of which are perceived by the public to pose a greater threat to people [1].

Worth emphasizing is also the fact that there are significant gender differences in the proportion of deaths worldwide caused by excessive alcohol consumption. According to 2012 data, up to 7.6% of deaths among men were attributed to alcohol, while for women, the rate was almost twice as low (4.0%). In many of these cases, determination of the exact cause of death is important for forensic medical opinion, and the circumstances of the death do not always allow to assess the effect of ethanol had on it. Only a routine toxicological analysis of biological material collected intra-sectionally and determination of blood ethanol concentration at a sufficiently high level (approximately 4‰ (g/L)) makes it possible to conclude that death was the result of acute alcohol intoxication [2].

In humans, the primary route for ethanol metabolism is hepatic oxidation, accounting for over 90% of ethanol elimination. Initially, ethanol is converted into acetaldehyde, primarily by cytosolic alcohol dehydrogenase, although enzymes such as cytochrome P450 isoforms—especially CYP2E1—and catalase also contribute to this step [3]. Subsequently, acetaldehyde is further oxidized to acetate by aldehyde dehydrogenase. The resulting acetate is transformed into acetyl–CoA by peripheral tissues, including the brain, heart, and skeletal muscle [4].

Apart from oxidative metabolism, ethanol also undergoes minor non-oxidative transformations through enzymatic conjugation with endogenous molecules like glucuronic acid, sulphate, phospholipids, and fatty acids. These reactions yield measurable metabolites such as ethyl glucuronide (EtG), ethyl sulphate (EtS), phosphatidylethanol (PEth), and fatty acid ethyl esters (FAEEs) [5]. While these non-oxidative routes account for a small fraction of total ethanol metabolism—ranging from approximately 1% in infrequent drinkers to 5% in chronic users—they are noteworthy due to the prolonged presence of their metabolites in tissues and biological fluids.

This characteristic feature makes non-oxidative ethanol metabolites amenable to retrospective assessment of ethanol intake, even when ethanol itself is no longer present in the body [3,6]. That is why they arepotential biomarkers for the assessment of ethanol intake with possible applications not only in health condition assessment, as well as in post-mortem studies [7]. Although ethanol metabolites are measured in forensics using various techniques—gas chromatography and liquid chromatography—in this study, we focused on gas chromatography coupled with mass spectrometry [6,8,9]. Although technique such as liquid chromatography might be also employed, this particular methodological combination provides superior analytical sensitivity and specificity for the detection and quantification of analytes of interest.

FAEEs are metabolites of the non-oxidative elimination pathway of ethanol that have been recognized as mediators of alcohol-induced organ damage. They are detectable in blood after ethanol ingestion and, therefore, are markers of ethanol consumption. FAEEs have also been quantified in human liver and adipose tissue—as potential post-mortem markers of ethanol consumption [10].

Most of the currently known diagnostic markers of chronic alcoholism have limited clinical utility. Distinguishing between a chronic alcohol abuser and an episodic heavy drinker (binge drinker) is possible by determining types or values of FAEEs’ concentrations in blood samples taken at two time points (at the onset of maximum blood ethanol concentration and approximately 24 h after ethanol withdrawal). FAEEs’ concentrations in blood and tissues might therefore be markers of ethanol intake and may be useful in distinguishing alcoholics from those who are occasionally taking high dose of alcohol. In addition, FAEEs can also be determined in meconium, confirming their utility in e.g., identifying chronic ethanol abuse in pregnant women [11,12].

Based on the literature, seven FAEEs were selected as the focus of this study. Their selection was based on specificity for the study group, frequency of occurrence, and the concentrations determined [13,14]. In forensic practice, it is almost impossible to completely avoid the blood samples’ irreversible hemolysis due to post-mortem changes. In order to minimize the impact of this factor, efforts were made to select the study group as accurately as possible. The post-mortem biological samples used in this study are extremely challenging, especially when it comes to the stability of the metabolites mentioned above. The QbD concept was applied during the development of an analytical method and to assess the influence of all method parameters on its performance. By using the QbD approach, it is possible to find, e.g., the optimal conditions to carry out analytical determinations or to obtain the highest recovery of analytes. Therefore, to overcome difficulties caused by the material of interest and to ensure method robustness, the design of experiments (DoE) approach was implemented. DoE involves assessing the influence of various factors on the selected outcome of an experiment. The key objective is to create an experimental plan that allows a thorough understanding of the relationships between input factors (process parameters) and output factors (experimental results), thereby enabling the experiment to be conducted in the most practical, usable, and satisfying way possible [15,16]. This is one of the first studies applying the DoE approach to establish the FAEEs’ determination protocol in post-mortem plasma samples.

## 2. Materials and Methods

### 2.1. Chemicals and Reagents

In order to perform the study, reagents listed below were used: deionized water obtained using Milli-RO and Milli-QPLUS apparatus (Millipore, Vienna, Austria), alkane standard mixture for GC analysis (Restek, Centre County, PA, USA), methanol for GC-MS (J.T. Baker, Deventer, The Netherlands), heptane for GC-MS (J.T. Baker, Deventer, The Netherlands isopropanol for LC-MS (J.T. Baker, Deventer, The Netherlands), ethyl laurate (Sigma-Aldrich, Schnelldorf, Germany), ethyl myristate (Sigma-Aldrich, Schnelldorf, Germany), ethyl palmitate (Sigma-Aldrich, Saint Louis, MO, USA), ethyl heptadecanoate (Sigma-Aldrich, Saint Louis, MO, USA), ethyl linoleate (Sigma-Aldrich, Saint Louis, MO, USA), ethyl oleate (Sigma-Aldrich, Saint Louis, MO, USA), and ethyl stearate (Sigma-Aldrich, Buchs, Switzerland).

### 2.2. Equipment

Gas chromatograph coupled with a mass spectrometer with electron ionization (EI) and triple quadrupole analyzer, GCMS-TQ8030 (Shimadzu, Kyoto, Japan), was used to perform the analytical determinations of plasma samples. Other instruments used in the study were as follows: Windows-based personal computers with the following software: GC/MS Solution Software 4.45 (Shimadzu, Kyoto, Japan), Statistica 13.3 (TIBCO Software Inc., Palo Alto, CA, USA), Microsoft Excel 2016 (Microsoft, Redmond, WA, USA), and JMP 17 Pro (JMP Statistical Discovery LLC., Campus Drive, Cary, NC, USA); Electrolux EJ2800AOW fridge–freezer (Electrolux, Stockholm, Sweden); ultrasonic bath (Polsonic, Warsaw, Poland); automatic pipettes (Eppendorf, Germany; Socorex, Switzerland); Milli-RO and Milli-QPLUS filter system (Millipore, Vienna, Austria); vacuum centrifuge with freeze trap and simultaneous evaporation of samples, Quatro MiVac Concentration (GeneVac, Suffolk, UK); MPW-260 R refrigerated laboratory centrifuge (MPW MED. Instruments, Warsaw, Poland); Eppendorf 5415 R refrigerated laboratory centrifuge (Eppendorf, Hamburg, Germany); laboratory shaker, MS 3 basic (IKA, Wilmington, NC, USA); Glacier Blue NU 966-8E freezer (NuAire, Plymouth, MN, USA); and Radwag XA/60/220/X laboratory balance (Radwag, Radom, Poland).

### 2.3. Preparation of Solutions

Stock solutions of each analyte were prepared by dissolving their appropriate weight in methanol in order to obtain a concentration of 1 mg/mL. Working solutions of each analyte were prepared from the solutions thus prepared. With these, samples were prepared in concentrations ranging from 0.015 µg/mL to 10 µg/mL. Calibration standards and QC samples were prepared from separate working solutions. In addition, a suitable internal standard was selected, namely heptadecanoic acid ethyl ester, which is a compound that does not occur physiologically in the body. It was added to each sample in a volume of 25 µL (100 µg/mL solution in methanol).

### 2.4. Plasma Sample Collection

Biological material for this study was obtained from the Department of Forensic Medicine, Faculty of Medicine, Medical University of Gdańsk during a lawful autopsy procedure. This study (including collection of plasma, liver and brain samples) was approved by the Independent Bioethics Committee for Scientific Research of the Medical University of Gdańsk (no. NKBBN/143/2019 dated 14 March 2019 and NKBBN/143-355,356/2019 dated 4 July 2019.). Both the study group and control group were matched in terms of age (women and men between 18 and 65 years of age, all other personal data has been anonymized) and time of autolysis (no longer than 96 h). Samples were also collected only from deceased with no recorded objection prior to death (based on Polish Act of 1 July 2005 on the Collection, Storage and Transplantation of Cells, Tissues and Organs, Journal of Laws 2020, item 2134, Article 5, presumed consent of the deceased applies unless an explicit objection was registered during their lifetime). The value of post-mortem whole blood alcohol concentration (BCA) was taken as the main differentiating factor between the two groups, where its level in the control group was below the limit of quantification, while in the study group, it was equal to or higher than 0.2‰.

For the method validation purpose, a mixture of six independent plasma samples obtained from healthy patients of Regional Centre for Blood Donation and Haemotherapy in Gdansk was used.

Real plasma samples were collected at two time points. The first plasma collection took place within 24 h of the presumed time of death, when the body was transported to a cold room (temperature 4–8 °C) where it remained pending autopsy. The body of the deceased was taken out of the cold room for material collection. Using a fifteen-centimetre chrome-plated brass needle, a puncture was made through the skin layers at the level of the supraclavicular fossa. Blood was collected in tubes containing heparin as anticoagulant and centrifuged for 15 min at 4 °C. The plasma obtained (collected from above the sediment of morphotic elements) was transferred into Eppendorf-type tubes and frozen at −80 °C. The needle was left in the body after collection. The needle tip was secured with a medical adhesive to avoid accidental slipping of the needle during subsequent autopsy procedures. A second plasma collection from the body of the same deceased took place during a scheduled autopsy. In order to avoid high variability in the study group due to the timing of material collection, autopsies of eligible patients were scheduled within 96 h of the presumed time of death. Material for examination was collected intersectionally after opening the body shell, directly from the left ventricle. At the same time, the puncture site used for material collection through the body shell during first time point was checked (to verify whether the first collection was made from the heart chambers, the aorta, or, for example, from the pericardial sac).

### 2.5. Plasma Sample Preparation

**Figure 1:**
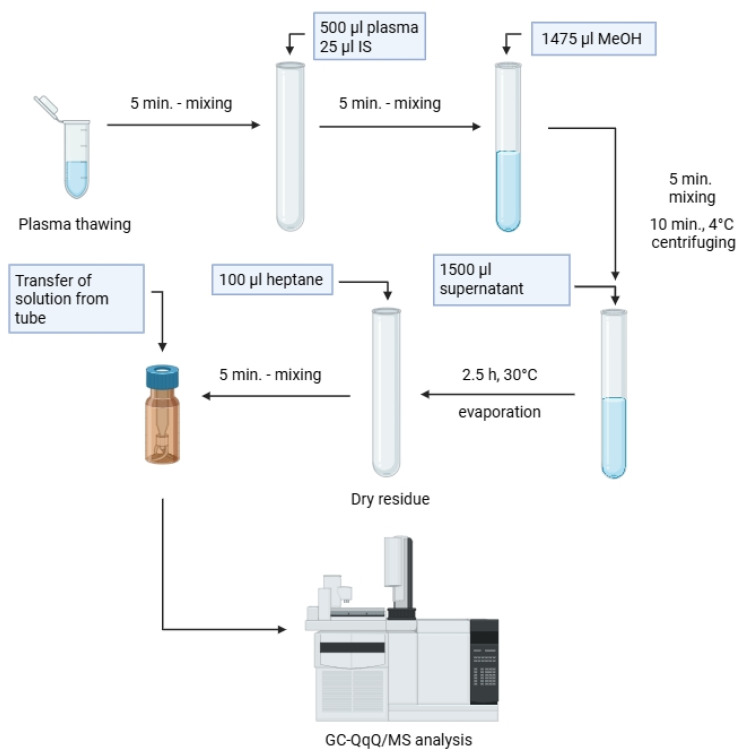
Graphical explanation of analytical procedure for plasma samples leading to GC-QqQ/MS analysis.

In the first step, plasma samples were thawed at room temperature and subjected to stirring in a laboratory shaker for 5 min. Then, 500 μL of plasma was transferred to 10 mL glass tubes, and 25 μL of previously prepared internal standard solution (heptadecanoic acid ethyl ester in methanol, 100 µg/mL) was added. The resulting mixtures were shaken for 5 min, after which 1475 μL of cold methanol (stored for 30 min at −80 °C) was added. The samples were mixed in a laboratory shaker for 5 min and then centrifuged for 15 min (2469× *g*) at 4 °C. In the next step, 1500 μL of the obtained supernatants were transferred to new 10 mL glass tubes and evaporated to dryness in a vacuum centrifuge with a freeze trap (30 °C; 2.5 h). 100 μL of heptane was added to the dry residues and stirred for 5 min using a laboratory shaker. The resulting solutions were transferred entirely into 200 μL glass inserts placed in 2 mL vials. The prepared samples were placed in a thermostated (11 °C) autosampler and analyzed by GC-QqQ/MS (Figure 1).

### 2.6. Method Development and Optimization

Analytical determinations of the prepared plasma samples were carried out using a gas chromatograph coupled with a mass spectrometer with a triple quadrupole analyzer with electron ionization (GC-EI-QqQ/MS) in MRM mode. Separation of the selected compounds was performed using a Zebron ZB-5MS chromatographic column (30 m × 0.25 mm, 0.25 μm; Phenomenex, Torrance, CA, USA). Given the physico-chemical properties of analytes, a wide range of temperature values and their gradient changes were tested. The parameters of the method for the determination of selected seven FAEEs in the biological matrix studied were optimized basing on those tests. The final temperature gradient programme is shown in Table 1. Prior to performing the analyses, the GC-QqQ/MS instrument was calibrated with a perfluorotributylamine (PFTBA) solution to ensure the quality of the measurements.

Starting with the sample preparation process—where evaporation of the solvent after extraction constitute a key step—the recovery of the analytes was checked. It was found that despite the longer time of the whole process compared to the usage of higher temperatures, the highest recovery was obtained for evaporation at 30 °C. Another parameter optimized was the volume ratio of the organic solvent to the biological matrix during the deproteinization step. A plasma/methanol volume ratio of 1:3 (*v*/*v*) proved to be the most favourable. Single analysis time was 57 min and 10 s. At this stage, the signals obtained were compared in terms of both, intensity and repeatability. The parameters of the optimized analytical method were set as follows: (1) sample injection volume—1 µL; (2) sample injection mode—splitless; (3) injection port temperature—250 °C; (4) carrier gas, flow, and pressure—helium, 10 mL/min, and 53.5 kPa; (5) ion source voltage and temperature—70 eV and 200 °C; and (6) CID gas—argon.

During the optimization of quantification method, the characteristic *m*/*z*, i.e., mass-to-charge ratios of fragment ions for the analytes to be determined, were selected (Table 2).

### 2.7. Method Validation

The analytical method developed was validated according to FDA and EMA guidelines for parameters such as linearity, selectivity and specificity, limit of detection (LOD) and limit of quantification (LOQ), precision, intermediate precision, accuracy (ACC), matrix effect, robustness, and analyte stability [17].

During this study, QC samples (QCs) were also analyzed. The procedure for the preparation of QCs was analogous to that for the preparation of real plasma samples.

**Figure 2:**
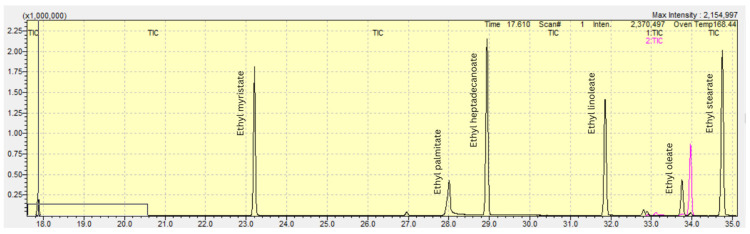
HQC sample analysis with the analytes’ peaks determined. Analysis carried out on a mixture of standard solutions.

The calibration curve, depending on the analyte being determined, included 7 or 8 measurement points at concentrations of 0.015, 0.03, 0.05, 0.25, 0.5, 2.5, 5.0, and 10 µg/mL. In addition, three QCs were prepared at low, medium, and high levels. Low quality control (LQC), medium quality control (MQC), and high quality control (HQC) samples were prepared by adding an appropriate volume of analytes’ mixture and internal standard to a blank sample in order to obtain final concentrations of 0.1 (LQC), 1.0 (MQC), and 7.5 µg/mL (HQC), respectively (Figure 2).

### 2.8. Robustness Analysis

Among the main applications of the Plackett–Burman plan is the optimization of chemical processes and improvement of their efficiency. It is used to analyze experiments where the number of variables is divisible by 4, and missing variables can be replaced by dummy variables (dummy factor), which serve to provide additional verification of the validity of analyses [18].

The Plackett–Burman plan is also often used as an initial step in the optimization process. It helps to identify the most important factors influencing the studied process. The impact of these factors can be evaluated in detail during further experiments, such as response surface methodology or factorial plans. It is therefore a useful tool for engineers and scientists involved in improving manufacturing and chemical processes, as well as verifying the robustness of an analytical method.

As previously mentioned, all of the analytes of interest are characterized by defined conditions (all the analytes of interest require very stringent conditions due to their stability during storage and analytical determinations). That is why it was very important to carefully set up right process variables. The parameters of sample preparation procedure, such as mixing time in laboratory shaker, temperature in laboratory centrifuge, volume of methanol used to precipitate the proteins in samples, and evaporation temperature, were tested in terms of their robustness.

In order to verify the robustness of the method, a series of 16 experiments was carried out to test the influence of small variations in parameters of different sample preparation steps on the final result of the determination. Plackett–Burman plan was selected as it is effective and is successfully used for robustness analysis [19,20,21].

Samples for the experiments were prepared according to the parameter values presented in Table 3. Reagents used in the experiments were the same as those used in the standard method of sample preparation.

## 3. Results

### 3.1. Method Validation

Six compounds, belonging to the FAEE group, were selected for quantification. However, during validation, it was decided not to continue with the quantification of ethyl laurate, as some of the validation parameters criteria were not met, including inter alia, the requirements for precision and stability. Unfortunately, it was not possible to obtain reproducible and reliable results for this compound during the simultaneous determination of the other compounds from FAEE group. Due to ethyl laurate’s chemical structure, it did not have sufficient stability for determination under the conditions of the method proposed, available equipment, and technique used. The available literature also shows that this compound is the most difficult to identify and accurately determine using the GC-MS technique [9]. Also, the concentration of ethyl laurate in collected biological samples would most likely be at levels below the limit of quantification. As it is not an endogenous compound, there are no data in the literature on average values of ethyl laurate in the human body.

#### 3.1.1. Selectivity and Specificity

**Figure 3:**
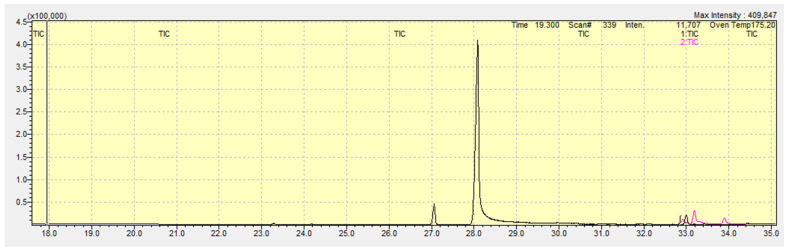
Total Ion Chromatogram (TIC) of the blank sample analysis with the visible IS peak (ethyl heptadecanoate at the 28th min.) X axis represents the retention time of analytes in minutes. Y axis represents the absolute abundance of analytes.

Assessment of the selectivity and specificity of the developed method was carried out by analyzing blank plasma samples prepared in the same way as the real biological samples analyzed subsequently. Blank samples were analyzed in each sequence. Figure 3 shows an example of analysis of blank sample.

During method validation, the absence of other compounds that could interfere with the measurement of the concentration of selected ethyl esters of fatty acids in the analyzed material was proven.

#### 3.1.2. Linearity, Limit of Detection, and Limit of Quantification

To determine LOD and LOQ values, plasma samples spiked with a mixture of standard analytes at low concentrations of 0.01, 0.015, 0.02, 0.03, 0.05, and 0.1 µg/mL were analyzed. Such analysis was performed twice, each time from newly prepared set of samples. Based on the ratio of the area of each analyte to the area of internal standard and nominal concentration, curves were determined, and LOD and LOQ values were calculated. The LOQ values determined by the low concentration curve method are shown in Table 4.

Analysis of samples of human plasma confirmed that each concentration range was selected correctly.

Each sequence started with the analysis of blank sample as well as blank sample with internal standard. Concentration values for ULOQ (upper limit of quantification) for each FAEE were selected based on a literature review. The determination of the LLOQ, on the other hand, was motivated by the expected very low concentrations in the biological samples tested, especially among the control group.

Samples from the test group were mostly within the linearity range of the method. In contrast, samples from the control group did not, in many cases, fall within the linearity range of the method. It was assumed, however, that this was not due to insufficient sensitivity of the developed method, but due to the nature of FAEE compounds, which, as metabolites of ethanol, are not physiologically present in the body.

The results obtained coincide with the values determined with the use of signal-to-noise ratio, i.e., LOQ = 0.015 µg/mL for ethyl laurate, myristate, palmitate, and stearate, and LOQ = 0.03 µg/mL for ethyl oleate and linoleate.

#### 3.1.3. Precision and Accuracy

To assess reproducibility, a set of 18 samples at concentrations of 0.1 µg/mL (*n* = 6), 1 µg/mL (*n* = 6), and 7.5 µg/mL (*n* = 6) was analyzed (i.e., LQC, MQC, and HQC). These determinations were performed on the same day, by the same investigator, in the same sequence. Using the coefficient of variation (CV), the reproducibility of the method was assessed. A CV value of no more than 10% for the five FAEEs provedthe precision of developed method.

In addition, the precision of the method was verified at LLOQ concentrations of 0.015 µg/mL for ethyl myristate, palmitate and stearate and 0.03 µg/mL for ethyl linoleate and oleate.

Furthermore, accuracy and precision were determined by comparing results from three different sequences on three separate days (at two-day intervals for 3 days) (Supplementary Materials, Table S1).

#### 3.1.4. Matrix Effect

For each analyte, a matrix factor (MF) was calculated by dividing the area under the peak of the analyte in biological matrix by the area under the peak of the analyte prepared at the same concentration in methanol solution. The MF was also calculated for the internal standard. The obtained matrix ratios for each analyte were then normalized by the MF of the internal standard. Furthermore, based on the obtained normalized matrix coefficients, coefficient of variation was calculated. The calculated coefficients of variation were not greater than 15%.

The internal standard (heptadecanoic acid ethyl ester), in both, biological matrix and methanol, was determined at very similar levels.

In most cases, it was confirmed that plasma as a biological matrix influences the calculated concentration of the determined compounds.

The results of the matrix effect calculations are presented in Table 5.

#### 3.1.5. Stability of Analytes

A stability assessment was carried out to ensure that each step of sample preparation procedure, as well as storage and analysis of the biological material, did not significantly affect the determined concentration of analytes. These parameters were checked using samples at LQC and HQC levels. Results of the stability tests are presented in Table S2 (Supplementary Materials).

(a) Freeze–thaw test

Three measurements of re-frozen samples were performed, proving that there was no effect of multiple (three cycles of freezing and thawing) freezing cycles on the concentration of the determined analytes.

(b) Stability during sample preparation

For each compound, the determined average concentration at HQC level ranged from 7.73 µg/mL (for ethyl palmitate) to 7.99 µg/mL (for ethyl myristate). At the LQC level, the determined mean concentrations were lowest for ethyl stearate (0.09 µg/mL), and the highest (0.12 µg/mL) in the case of ethyl palmitate. Thus, it can be concluded that the sample preparation process of almost 20 h has no significant effect on the determinations of FAEEs in the biological samples analyzed.

(c) Stability in the autosampler

After preparation, QCs were placed in the autosampler and were subjected to analysis—half of the set immediately and half after 48 h. The temperature of 11 °C, which was maintained in the autosampler, reduced solvent evaporation from the prepared plasma samples and increased the stability of the analytes.

Summing up the results of the stability assessment, it is recommended to divide the collected biological material into smaller portions in order to avoid unnecessary thawing and freezing processes. However, in cases where material has already been preserved in larger amounts, there is no need to abandon or limit its use, as the effect of the thawing and freezing process on the concentration of analytes in the samples appeared to be negligible. The effect of storing samples under laboratory conditions during the preparation process and finally holding them in the autosampler during the sequence run is not significant.

### 3.2. Robustness of the Analytical Method

The robustness of the analytical method developed was confirmed for four (ethyl palmitate, linoleate, oleate, and stearate) out of five FAEEs determined. In the case of ethyl myristate, the variable significantly affecting the results appeared to be the temperature of solvent evaporation after deproteinization. A difference of one degree Celsius results in an approximately 11% decrease in the content of ethyl myristate determined. In the case of the other determined compounds, no such relationship was observed (Table 6).

**Figure 4:**
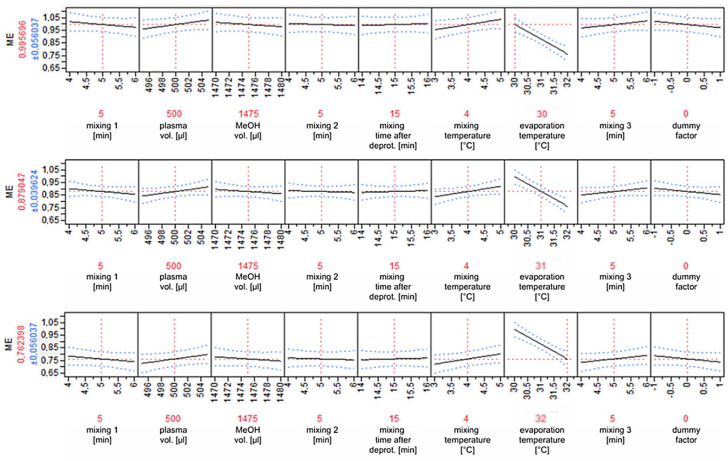
Comparison of built model predictions for 3 different solvent evaporation temperatures for ethyl myristate determinations (top: 30 °C; middle: 31 °C; bottom: 32 °C).

The graphical representation of relationships between variables clearly illustrates that strict control of the evaporation temperature is a key step ensuring reproducibility of the analyses, and any deviations in that parameter significantly influence the results obtained in the form of the values of ethyl myristate concentrations present in the biological samples (Figure 4).

The other parameters verified were not characterized by a significant influence on the results of the analytical determinations carried out.

### 3.3. Analysis of Real Plasma Samples

As a result of preliminary analyses of the collected plasma, it was concluded that the plastic tubes should be changed to those that are not subjected to high temperatures in the autoclave. The autoclaved tubes were a source of plasticizers that prevented proper analysis of the chromatograms obtained. After changing the tubes to non-sterilized ones, the source of interfering signals was eliminated.

Finally, biological samples from 31 cases were qualified for FAEEs quantification. These fulfilled the inclusion and exclusion criteria, and their volume and quality made the experiments feasible. The inclusion and exclusion criteria were as follows: (1) age between 18 and 64; (2) autolysis time lower than 96 h; (3) post-mortem blood alcohol concentration ≥ 0.2‰ (test group) or below LOQ (control group); (4) exclusionary medical conditions: mental disorders or organic diseases of neurodegenerative and psychiatric origin and organic changes in internal organs of infectious, oncological, and extremely infectious aetiology; and (5) exclusionary circumstances: death while consuming alcohol.

Plasma samples subjected to analysis were randomly divided into two groups that were analyzed in separate sequences. Plasma samples were analyzed in random order, and a quality control sample (LQC, MQC, or HQC) was analyzed every six injections. Consistent conditions were maintained throughout the analyses, including, but not limited to, a temperature in the autosampler of 11 °C.

The analyses resulted in the determination of FAEEs concentrations in samples from 31 patients taken at two time points: (1) within 24 h of the presumed time of death (through body shells) and (2) at the time of autopsy (directly from the heart). The verage concentrations of determined metabolites are presented in Table 7.

The analyses confirmed the correct selection of the concentration range of the calibration curves. In most cases, the concentrations of the analytes calculated for the samples were within the range of the calibration curve. In numerous cases of the control group, the level of the FAEEs determined was below the limit of quantification, which was due to the characteristics of the samples classified in this group. Despite the small size of the groups, an attempt was made to determine the discriminatory value of the determined esters. The relationship between the concentrations of the determined metabolites and the post-mortem alcohol level in whole blood was assessed, and the distribution of each compound in the samples tested was checked. As the conditions for parametric tests (e.g., normality of distribution) were not met, a non-parametric method (Mann-Whitney U test) was used. The differences in the concentrations of the determined compounds in patients of both groups were statistically significant (*p* < 0.05).

Based on the obtained concentration values, a principal component analysis (PCA) was carried out using Statistica (14.0.1.25). This method is most commonly used for data matrix reduction, preliminary evaluation of analytical data, and the search for correlations between variables. The model built describes the actual data well. Thus, it was observed that without indication of the study group, the model is able to assign the evaluated case to the appropriate group: control or study on the basis of the FAEEs concentrations.

**Figure 5:**
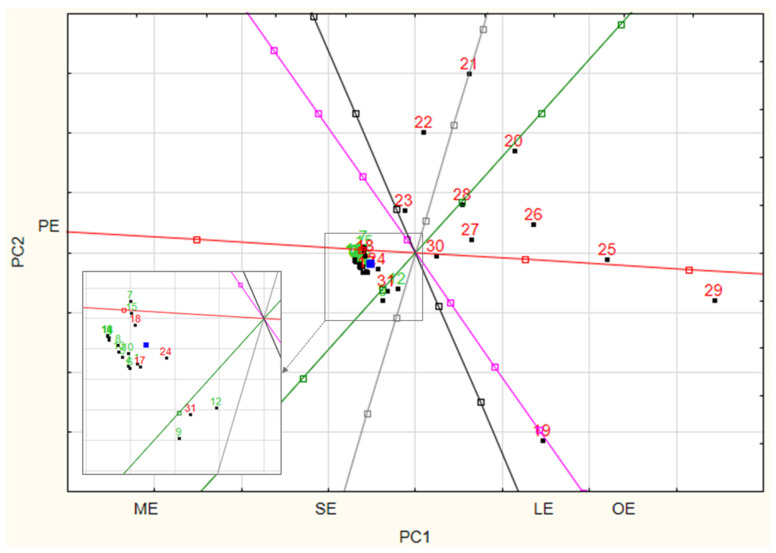
Standardized biplot (PC1 vs. PC2) of PCA for data from quantification of FAEEs; ME—ethyl myristate; SE—ethyl stearate; LE—ethyl linoleate; OE—ethyl oleate; PE—ethyl palmitate. Control group—green data points, test group—red data points.

As a result of the analyses of the two component variables, it can be concluded that, among the determined compounds, a strong correlation is observed between ethyl myristate and ethyl stearate, as well as between ethyl linoleate and ethyl oleate. For ethyl stearate and ethyl oleate, on the other hand, as well as ethyl linoleate and myristate, the angles were approximately 90°, which may mean that the information they provide is independent of each other. It was also observed that samples from patients who are controls (sober at the time of death) form a clustered group, while samples from those under the influence of alcohol at the time of death are characterized by a greater scatter (Figure 5).

## 4. Discussion

Due to the unique nature of the research carried out by toxicologists and forensic medics, the toxicological analyses widely used in forensic medicine must be constantly monitored and improved in order to not only obtain more accurate results but also to obtain them as quickly as possible. In forensic analysis, the material for testing can be selected from a wide range of tissues. The following study focuses on plasma samples from deceased individuals. Despite the fact that this is a widely known, used, and relatively uncomplicated matrix, in this case, the usefulness of most of the material secured has been questioned due to the haemolysis process and the small blood volume.

The problems encountered at the plasma preservation stage were surprising and often impossible to predict. (1) The first plasma samples, after centrifugation, were transferred to Eppendorf-type tubes, which had previously undergone an autoclaving process (a procedure regularly carried out in the Department of Forensic Medicine in order to make the tubes useful for securing material for genetic analyses). In the case of the analyzed esters, this method did not work because of the released plasticizers, which strongly interfered with MS-based analyses. (2) Another obstacle, difficult to foresee, was the aforementioned haemolysis and the availability of blood for collection. On more than one occasion, a case initially eligible for inclusion in one of the groups had to be rejected because the person had died of internal haemorrhage and the volume of blood available for collection was too low, if not non-existent. In many cases—even those already qualified for analysis—the available plasma volume only allowed for a single targeted analysis. The extent of hemolysis can vary and is influenced by factors like the time since death, temperature, and individual factors. This makes it difficult to standardize and interpret results reliably. This fact significantly influenced the choice of the method of sample preparation for analysis, as well as the analysis itself (the original assumptions were that group sizes would be at least twice as high as the final ones used for this study).

A study of the robustness of the analytical method used highlighted the importance of controlling various stages of the sample preparation process, in particular, the solvent evaporation temperature. (3) Due to the thermolability of the analytes being determined, it is important to keep the temperature as low as possible during the sample preparation process and consequently, rather low during storage in the autosampler. The solvent evaporation step after plasma deproteinization was found to be crucial. (4) Due to apparatus limitations, the minimum temperature that can be applied and controlled throughout the process was 30 °C. Vacuum concentrators that can also be used at lower temperatures are available from laboratory equipment companies, but their usage was not possible in this study. It is reasonable to assume that lowering the solvent evaporation temperature by a few degrees Celsius will have an even more favourable effect on the recovery of the analytes determined from the dry residue, as well as on the repeatability of the results, particularly for ethyl myristate. The high sensitivity of the ethyl esters analyzed to temperature conditions and their considerable volatility may be due to the chemical bonds they form. They participate in hydrogen bonds as hydrogen bond acceptors, but cannot act as hydrogen bond donors, unlike their parent alcohols. This ability to participate in hydrogen bonding gives them a certain solubility in water. Due to their inability to donate hydrogen bonds, esters do not self-associate. Consequently, esters are more volatile than carboxylic acids of similar molecular weight [22].

When analyzing the results of individual FAEEs in human plasma collected post-mortem, it can be concluded that the selected compounds may be potential indicators of excessive ethanol consumption. As confirmed in other studies focusing on FAEEs, they persist at elevated levels in the body for several tens of hours after consumption [23,24].

Of all the FAEEs determined in the following study, it is oleate that is most likely to reflect the amount of alcohol at the time prior to death (even though the post-mortem examination did not reveal the presence of alcohol in the blood), which was also confirmed in the work by Soderberg and colleagues [25]. The results of their study suggested that a concentration of ethyl oleate of 0.0004 µg/mL was a sign of chronic alcoholism. For the study group, such or higher concentrations were present in 96.67% and as high as 59.38% of cases in the control group. Ethyl stearate levels, on the other hand, appear to best reflect recent ethanol consumption, which was also confirmed by the standard whole blood examination.

## 5. Conclusions

Based on the determinations carried out in this study, it can be concluded that targeted metabolomic analysis of plasma samples is a useful research tool for assessing metabolic changes associated with ethanol consumption. Especially, the determination of FAEEs in post-mortem plasma samples is useful to confirm or exclude the presence of exogenous ethanol in the body. The application of the GC-QqQ-MS technique has allowed the development and validation of a robust method for the quantification of selected FAEEs in plasma samples from deceased individuals.

Principal component analyses showed that the determined concentration of ethyl stearate highly differentiates the collected samples, showing differences between the samples of the control and test groups.

The biochemical interpretation of the results obtained with the available medical records suggests that plasma FAEEs concentrations seem to be promising indicators of pre-mortem alcohol consumption and may be one of the factors helpful in determining the cause and mechanism of death.

## Figures and Tables

**Table 1:** Temperature gradient during GC-QqQ/MS determinations.

Temperature Change Rate [°C/min]	Final Temperature [°C]	Temperature Holding Time [min]
-	60	1.0
10.0	130	0.0
4.0	180	0.0
3.0	250	0.0
6.0	300	5.0

**Table 2:** Selected values for *m*/*z* transitions and collision energy (CE) of FAEEs determined by the developed method.

Analyte	Transition Type	Precursor Ion → Product Ion *m*/*z*	CE [V]
Ethyl laurate	quantifier	185 → 87	12
	qualifier	185 → 157	6
Ethyl myristate	quantifier	213 → 87	12
	qualifier	213 → 185	6
Ethyl palmitate	quantifier	241 → 87	12
	qualifier	213 → 87	24
Ethyl linoleate	quantifier	263 → 67	27
	qualifier	263 → 81	18
Ethyl oleate	quantifier	222 → 67	27
	qualifier	180 → 67.10	24
Ethyl stearate	quantifier	269 → 87	12
	qualifier	312 → 87	24

**Table 3:** Experiments carried out to test the robustness of the analytical method.

No	Mixing 1 [min]	Plasma Volume [µL]	MeOH Volume [µL]	Mixing 2 [min]	Centrifuging after Deprot. [min]	Mixing Temperature [°C]	Evaporation Temperature [°C]	Mixing 3 [min]	Dummy Factor
1	5	500	1475	5	15	4	30	5	0
2	5	500	1475	5	15	4	30	5	0
3	6	495	1470	4	16	3	30	6	−1
4	4	495	1480	4	16	5	32	4	−1
5	6	495	1480	6	16	3	30	4	1
6	4	505	1470	4	16	3	32	6	1
7	5	500	1475	5	15	4	32	5	0
8	4	505	1470	6	16	5	30	4	−1
9	6	495	1470	6	14	5	32	6	−1
10	6	505	1470	4	14	5	30	4	1
11	4	505	1480	6	14	3	30	6	−1
12	5	500	1475	5	15	4	32	5	0
13	4	495	1470	6	14	3	32	4	1
14	4	495	1480	4	14	5	30	6	1
15	6	505	1480	4	14	3	32	4	−1
16	6	505	1480	6	16	5	32	6	1

**Table 4:** Determined LOQ values of the analyzed compounds.

Analyte	Standard Error of a Mean	Standard Deviation	Calculated Value LOQ
Ethyl mirystate	0.00043	0.2845	0.0153
Ethyl palmitate	0.00040	0.2766	0.0144
Ethyl linoleate	0.00012	0.0404	0.0308
Ethyl oleate	0.00022	0.0908	0.0246
Ethyl stearate	0.00025	0.1771	0.0141

**Table 5:** Matrix effect calculations.

Analyte	Control Sample	$\frac{{MF}_{analyte}}{{MF}_{IS}}$	${CV}_{MF}$
Ethyl myristate	LQC	1.7867	7.14
	HQC	1.1270	1.70
Ethyl palmitate	LQC	1.2159	10.68
	HQC	0.9383	1.07
Ethyl linoleate	LQC	1.1086	2.31
	HQC	1.0373	8.29
Ethyl oleate	LQC	0.9596	10.72
	HQC	0.9856	9.40
Ethyl stearate	LQC	0.9383	1.07
	HQC	0.9001	4.48

**Table 6:** Variables assessed in the model for ethyl myristate.

Variable	Baseline	Average	Standard Deviation	Probability
Evaporation temp. [°C]	30	−0.1166	0.0162	0.0004
Centrifugation temp. [°C]	5	0.0407	0.0187	0.0726
Plasma volume [μL]	500	0.0354	0.0187	0.1072
Mixing 3 [min]	5	0.0278	0.0187	0.1871
Dummy variable	-	−0.0249	0.0187	0.2310
Mixing 1 [min]	5	−0.0218	0.0187	0.2881
MeOH volume [μL]	1500	−0.0173	0.0187	0.3908
Mixing 2 [min]	5	−0.0076	0.0187	0.6991
Centrifugation time [min]	15	0.0073	0.0187	0.7096

**Table 7:** Average concentrations of the analytes determined as a function of post-mortem blood ethanol concentration.

	Average Analyte Concentration ± Standard Deviation				
BAC [‰]	≤0.25	0.26–1.00	1.01–2.00	2.01–3.00	≥3.01
Group Size	1	2	2	3	7
Ethyl myristate	0.0064	0.0343 ± 0.048	0.2870 ± 0.254	0.1911 ± 1.321	4.2900 ± 0.292
Ethyl palmitate	0.0463	0.9603 ± 1.299	0.9836 ± 0.281	1.3395 ± 1.321	0.1643 ± 1.687
Ethyl linoleate	0.0266	1.8380 ± 2.461	0.3489 ± 0.406	0.9275 ± 1.296	1.8433 ± 1.256
Ethyl oleate	0.4419	1.9703 ± 2.405	1.0634 ± 1.116	1.6037 ± 1.844	1.0070 ± 1.195
Ethyl stearate	0.0189	0.2820 ± 0.244	1.1166 ± 0.147	0.5367 ± 0.436	3.0053 ± 0.442

## Data Availability

The raw data supporting the conclusions of this article will be made available by the authors on request.

## Supplementary Materials

The following supporting information can be downloaded at: https://www.mdpi.com/article/10.3390/biomedicines13071688/s1. Table S1: Results of reproducibility tests. Table S2: Stability tests’ results.
