# Supplementary material for: Overcoming Challenges in the Determination of Fatty Acid Ethyl Esters in Post-Mortem Plasma Samples with the Use of Targeted Metabolomics and the Quality by Design Approach
Source: Biomedicines. 2025 Jul 10;13(7):1688. doi: 10.3390/biomedicines13071688 (PMC12292823; doi:10.3390/biomedicines13071688)
Supplement: Supplementary file 1 [file biomedicines-13-01688-s001.zip › biomedicines-3700300-supplementary.pdf]

Table S1. Results of reproducibility tests.

Analyte	Control sample	Nominal concentration [µg/ml]	Day	intraday analysis [n=5] ACC [%]	intraday analysis [n=5] CV [%]	interday analysis [n=15] ACC [%]	interday analysis [n=15] CV [%]
**Ethyl Mirystate**	LLOQ	0.015	1	110.58	7.47	-	-
	LQC	0.1	1	109.03	4.39	107.36	6.81
			2	106.10	10.11		
			3	107.22	4.96		
	MQC	1	1	102.19	8.97	104.86	7.19
			2	108.56	5.83		
			3	104.35	6.07		
	HQC	7.5	1	105.11	9.75	102.01	8.12
			2	104.27	7.04		
			3	96.65	5.37		
**Ethyl Palmitate**	LLOQ	0.015	1	110.10	4.61	-	-
	LQC	0.1	1	90.50	6.22	97.4	8.33
			2	98.20	3.03		
			3	104.76	8.15		
	MQC	1	1	102.41	6.43	101.24	5.10
			2	100.66	3.54		
			3	102.82	3.08		
	HQC	7.5	1	101.13	1.70	101.47	3.68
			2	102.63	5.76		
			3	100.57	2.14		
**Ethyl Linoleate**	LLOQ	0.03	1	98.25	6.60	-	-
	LQC	0,1	1	111.74	0.86	100.62	8.36
			2	93.00	4.19		
			3	99.98	3.73		
	MQC	1	1	101.48	3.82	102.58	3.55
			2	101.26	2.44		
			3	105.55	2.67		
	HQC	7.5	1	93.87	4.46	95.82	4.80
			2	99.03	5.91		
			3	95.52	1.86		
**Ethyl Oleate**	LLOQ	0.03	1	112.06	3.22	-	-
	LQC	0.1	1	99.22	5.65	96.89	7.97
			2	96.65	10.91		
			3	94.32	7.97		
	MQC	1	1	102.10	3.36	103.72	2.99
			2	103.70	2.09		
			3	105.65	2.60		
	HQC	7.5	1	94.68	5.29	97.83	8.89

			2	102.04	6.08		
			3	98.36	1.09		
**Ethyl Stearate**	LLOQ	0.015	1	103.45	8.76	-	-
	LQC	0.1	1	105.54	3.94	105.65	5.82
			2	108.90	8.44		
			3	102.53	2.53		
	MQC	1	1	96.51	6.38	102.78	8.03
			2	102.21	1.40		
			3	109.72	1.99		
	HQC	7.5	1	99.33	7.35	105.04	8.70
			2	110.05	4.19		
			3	106.58	5.00		

Table S2. Stability tests' results.

Analyte	QC level	Nominal concentration [µg/ml]	Stability during sample preparation (n=3)	Stability in the autosampler (n=3)	Freeze-thaw stability		
			3h [%]	48 h [%]	cycle 1 [%] (n=3)	cycle 2 [%] (n=3)	cycle 3 [%] (n=3)
**Ethyl myristate**	LQC	0.015	110.08	90.11	86.72	90.03	102.02
	HQC	7.5	100.09	96.63	90.30	98.30	113.64
**Ethyl palmitate**	LQC	0.015	111.64	92.49	89.78	89.99	88.85
	HQC	7.5	104.49	103.02	91.63	102.70	96.67
**Ethyl linoleate**	LQC	0.015	101.54	88.93	88.05	87.78	87.30
	HQC	7.5	93.79	90.97	90.66	97.12	97.79
**Ethyl oleate**	LQC	0.015	107.02	99.96	92.87	93.44	94.19
	HQC	7.5	98.55	101.34	96.25	103.98	99.92
**Ethyl steareate**	LQC	0.015	108.20	89.19	97.65	94.65	89.88
	HQC	7.5	100.09	96.64	96.38	107.18	99.24
